# Femtosecond laser assisted phacoemulsification plus IOL implant: toric IOL vs. astigmatic keratotomy

**DOI:** 10.3389/fmed.2026.1752650

**Published:** 2026-04-17

**Authors:** Howard Wen-Haur Chao, Windsor Wen-Jin Chao, Cheng-Kuo Cheng, Shiow-Wen Liou, Pai-Huei Peng, Hsiao-Ming Chao

**Affiliations:** 1 Department of Medicine, School of Medicine, Aston University, Birmingham, United Kingdom; 2 Department of Medical Education, Leeds University, Leeds, United Kingdom; 3 Queen Elizabeth University Hospital, Birmingham, United Kingdom; 4 New Cross Hospital, Wolverhampton, United Kingdom; 5 Department of Science, University of British Columbia, Vancouver, BC, Canada; 6 Department of Ophthalmology, Shin Kong Wu Ho-Su Memorial Hospital, Taipei, Taiwan; 7 School of Medicine, Catholic Fu-Jen University, Taipei, Taiwan; 8 School of Medicine, National Taiwan University, Taipei, Taiwan; 9 Department of Chinese Medicine, School of Chinese Medicine, China Medical University, Taichung, Taiwan; 10 Institute of Pharmacology, School of Medicine, National Yang Ming Chiao Tung University, Taipei, Taiwan

**Keywords:** astigmatic keratotomy, corneal higher-order aberrations, Double Angle Vector Diagrams, femtosecond laser-assisted cataract surgery, ocular and corneal astigmatism, polar plots, toric intraocular lens, uncorrected distant visual acuity LogMAR

## Abstract

**Purpose:**

To compare the clinical outcomes of femtosecond laser-assisted cataract surgery (FSLACS) with astigmatic keratotomy (AK) versus toric intraocular lens (IOL_t_) implantation for astigmatism correction.

**Design:**

Retrospective comparative case series.

**Methods:**

60 eyes (45 IOL_t_ cases and 15 AK cases) with patient’s mean age of 61.46 ± 12.84 years underwent FSLACS with astigmatic correction. Pre and post-operative evaluations included autorefractometry, corneal topography, IOL Master analysis, and assessment of total ocular astigmatism (OA_tot_), anterior/posterior/total corneal astigmatism (CA_ant_/CA_post_/CA_tot_), corneal higher-order aberrations (HOAs), vector analysis, and best-corrected/uncorrected distant visual acuity (BCVA; UDVA; LogMAR). The average follow-up period was 16.29 ± 11.15 months.

**Results:**

In term of OA_tot_ as demonstrated by polar plots, significant reductions were observed in both the IOL_t_ and the AK groups (*p* < 0.001). In regard to CA_ant_, a statistically significant change was observed in the AK group. No significant change was noted in the IOL_t_ group. In view of CA_post_, there was no statistically significant difference in CA_post_ for either the IOL_t_ or AK groups, indicating stability of the posterior corneal surface. As for CA_tot_, the AK group showed a significant pre to post-operative change (*p* < 0.001). No significance was found in the IOL_t_ group. Likewise, corneal HOAs remained stable in IOL_t_ but significantly increased in AK (*p* < 0.01). This is associated with a significant intergroup difference of *p* < 0.001. Vector astigmatism analysis utilizing Double Angle Vector Diagrams to show that TIA/SIA/DV/CI for the IOL_t_ group and for the AK group. Vector astigmatism analysis implied that there was no significant difference in target/surgery induced astigmatism (TIA/SIA) between groups. BCVA (LogMAR) improved significantly in both groups [IOL_t_: 0.40 (0.40, 0.50) to 0.10 (0.00, 0.10); AK: 0.40 (0.40, 0.50) to 0.10 (0.10, 0.15); both *p* < 0.001]. There is with no significant difference between both groups (*p* = 0.49).

**Conclusion:**

FSLACS with toric IOL implantation offers an effective and more stable astigmatism correction without increasing HOAs, but also having similar visual outcome improvement, when compared to AK’s astigmatism correction.

## Introduction

In treating corneal astigmatism (CA) less than 1 diopter (D), manual astigmatic keratotomy (AK) is not inferior to toric intraocular lens (IOL_t_) implantation. Achieving precision in cut length, depth, and axis alignment is crucial for enhancing reproducibility and minimizing regression (1). Femtosecond laser-assisted (FSLA) AK (FSLAAK) offers distinct advantages over manual AK, including precise control over incision parameters (length, depth, and axis), increased surgical safety, and reproducible outcomes in cataract operations involving corneal astigmatism correction (2).

Nomograms have been developed to optimize visual outcomes using FSLA arcuate incisions (AIs) for correcting CA. Cataract patients with CA between 0.5 and 1.0 D are considered ideal candidates for FSLA cataract surgery (FSLACS) with AK (3). Studies have demonstrated that FSLAAK can provide safe and long-term reductions in low-to-moderate astigmatism—up to 2.5 D—in anterior corneal curvature and total corneal refractive power (4).

While AK is effective for lower levels of astigmatism, toric IOLs (IOL_t_) offer a valuable alternative, particularly in patients with higher degrees of astigmatism. IOL_t_s are especially capable of correcting CAs greater than 2.0 D and also avoiding the need for additional corneal incisions. However, the drawbacks include the risk of IOL_t_ rotation, where significant misalignment (over 10 degrees) may necessitate surgical repositioning of the lens.

This paper is a retrospective study and the primary outcome is to compare the efficacy of FSLACS with AK versus FSLACS with IOL_t_ for treating astigmatism up to 3.0 D. The study assesses changes in anterior corneal astigmatism (CAant), posterior corneal astigmatism (CApost), total corneal astigmatism (CAtot), total ocular astigmatism (OAtot), surgery induced astigmatism (SIA), correction index (CI), and best corrected visual acuity (BCVA; LogMAR). Secondary aim of this study is to evaluate the effects FSLACS with AK and who received FSLACS with IOL_t_ implantation on corneal wavefront, namely high order aberrations (HOAs), and cumulative dissipated energy (CDE).

## Methods

This retrospective comparative study included patients who underwent cataract surgery with either FSLAAK or IOL_t_ between July 2022 and July 2023 at the Department of Ophthalmology, Shin Kong General Hospital, Taipei (registration number: 220615R). Inclusion criteria include patients with age >40 years, corneal astigmatism (CA) ≤ 3.0 D, and cataract (nuclear cataract scale 3–4 by Lens Opacities Classification System III) necessitating surgery. Exclusion criteria included corneal pathology, irregular astigmatism, prior refractive surgery, ocular trauma, and glaucoma. A total of 60 eyes (FSLAAK: *n* = 15; IOL_t_: *n* = 45) had complete follow up data over 16.29 ± 11.15 months including IOL Master biometry, corneal topography, and best corrected visual acuity (BCVA; LogMAR). Each eye was recruited from one patient. The study adhered to the Declaration of Helsinki and received approval from the hospital’s Institutional Review Board. Finally, written inform consent has also been obtained from the patients.

### Pre-operative assessment

Prior to the surgery, all of the patients underwent ophthalmic biometry (IOL Master 700; Carl Zeiss Meditec, Jena, Germany), non-contact tonometry, Placido-based corneal topography (ATLAS; Carl Zeiss Meditec), and autorefractometry (VISUREF 150; Carl Zeiss Meditec). Measurements also included central corneal thickness (CCT; Crystalvue, CE1639, Crystalvue Medical Co., Taoyuan, Taiwan), endothelial cell density (ECD; Specular microscope, sp-3000p, Topcon, 75–1, Hasunuma-cho, Itabashi-ku, Tokyo 174–8,580, Japan), CA_ant_, CA_post_, and CA_tot_, OA_tot_, difference vector (DV), CI, TIA and SIA.

### Surgical planning and procedure

FSLACS-AK was programmed using a standardized nomogram (Catalys, Johnson & Johnson) with an 8-mm optical zone, arc length of 85 degrees and 80% corneal depth, which is also based on OA_tot_, CA_ant_, CA_post_, and CA_tot_.

Two reference points were marked at the slit lamp, then arcuate incisions were created with the femtosecond laser after docking, capsulotomy, and lens fragmentation. A temporal 2.2-mm limbal incision and 1-mm paracentesis were also created. Phacoemulsification was performed using WHITESTAR SIGNATURE PRO (Johnson & Johnson) or Centurion Vision System (Alcon, United States). The CDE values in torsional and longitudinal phacoemulsification modes were automatically calculated by the device and displayed on the monitor of the phacoemulsification system. Cortical aspiration was followed by non-toric IOL implantation. In the IOL_t_ group, a toric IOL (Tecnis Eyhance Toric II, Johnson & Johnson Vision, United States) was implanted. All surgeries were performed by the same surgeon. After the surgery, a standard postoperative regimen of topical antibiotics and steroids tapered over 4 weeks. Post-operative ocular examinations included BCVA, slit-lamp examination, ECD, intraocular pressure, CCT, autorefractometry, and corneal tomography were performed. HOAs and vector analysis (CI and SIA) using the Alpins Method were reassessed at 3 months.

Vector analysis with the Alpins method was performed to determine post-operative changes in CAant, CApost, and CAtot (5). The target-induced astigmatism vector (TIA), defined as the astigmatic change the surgery was intended to induce, was not equal to the preoperatively measured CAant, CApost, and CAtot, as the targeted residual astigmatism was defined case-wise to be between 0.3 and 0.5 D after surgery. Additionally, the magnitude of error, the DV, and the surgically induced astigmatism vector (SIA) were calculated, which define the induced astigmatic change that would enable the initial surgery to achieve its intended target and the amount and axis of astigmatic change the surgery actually induced, respectively. The absolute angle of error, described by the vectors of achieved correction (SIA) vs. the intended correction (TIA), was also calculated.

Polar plots were generated using ASTIGMATIC software (Gauvin & Wallerstein) (5). Double-angle plots were generated using the Astigmatism Double Angle Plot Tool (American Society of Cataract and Refractive Surgery) (6).

### Astigmatism classification and aberration analysis

Astigmatism was categorized as with-the-rule (67.5°–112.5°), against-the-rule (0°–22.5° or 157.5°–180°), or oblique (intermediate). At baseline (IOLt vs. AK), 13 *vs* 6 eyes had WTR, 21 *vs* 2 eyes had ATR, and 11 *vs* 7 eyes had oblique astigmatisms. High-order aberrations (HOAs) were measured over a 6-mm zone with ATLAS corneal topography and analysed using Zernike coefficients up to the 4th order. The root mean square value of total HOAs was calculated from 3rd- and 4^th^-order coefficients.

### Statistical analysis

Statistical analyses were performed using SPSS Version 23 (IBM). If the normality test and the Equal Variance Test passed, the outcomes were presented as mean±standard deviation (SD). If not, the Rank Sum Test begun and the results were expressed as median and quartiles, namely median [Q1, Q3]. Pre- and post-operative data were compared using paired *t*-tests, whereas between-group comparisons utilize independent *t*-tests or Mann–Whitney U tests. A *p* value < 0.05 was considered statistically significant.

Analysis of covariance (ANCOVA) was used to compare post-operative outcomes between groups while adjusting for baseline values as covariates to account for potential baseline differences. Because this study was retrospective and the group sizes were unequal, standardized mean differences (SMDs) were additionally calculated to assess baseline balance between groups. SMD values <0.1 were considered to indicate negligible imbalance, whereas higher values suggested increasing degrees of imbalance.

## Results

### Demographics

As mentioned previously, sixty eyes from patients were utilized in this investigation. The baseline refraction status (Table 1; mediuan [Q1, Q3]) was 1.75 (1.25, 2.25) D in IOL_t_ (range: 1.0–4.25 D) vs. 2.25 (1.50, 3.00) D in AK (range: 1.25–3.25 D), whcih was not significantly (*p* = 0.06) different between two groups. No cases of corneal perforation were observed. The mean follow-up period was 16.29 ± 11.15 months (Table 1; mediuan [Q1, Q3]); IOL_t_ vs. AK = 18.00 (6.20, 24.35) vs. 12.40 (12.00, 15.00); not significantly different with *p* = 0.06) after surgery.

**Table 1:** Baseline characteristics.

Parameters	Pre—FSLACS-AK (*n* = 15)	Pre—FSLACS-IOL_t_ (*n* = 45)	*p*
Ocular astigmatism (OA)	2.25 (1.50, 3.00)	1.75 (1.25, 2.25)	0.06
CA_tot_	1.79 ± 0.74	1.59 ± 0.63	0.32.
UDVA, median (Q1, Q3)			
LogMAR	0.60 (0.50, 1.00)	0.60 (0.40, 1.30)	0.74
Snellen equivalent	0.25 (0.10, 0.32)	0.25 (0.05, 0.40)	0.76
Age (y), median [Q1, Q3]	60.00 (53.00, 67.00)	63.00 (55.00, 69.50)	0.70
Sex, % (No)			
Female/Male	11 (73)/4 (27)	26 (58)/19 (42)	0.29
Eye laterality, No. (%)			
Right/Left	8 (53)/7 (47)	49 (22)/51 (23)	0.78
F-U (month), median [Q1, Q3]§	12.40 (12.00, 15.00)	18.00 (6.20, 24.35)	0.06
Preoperative astigmatism, No. (%)			
<1.5 D	5 (33)	17 (38)	
≥1.5 D	10 (67)	28 (62)	
Astigmatism type, No. (%)			
WTR/ATR/Oblique	6/2/7	13/21/11	
	(40/13/47)	(29/47/24)	

BCVA, best corrected visual acuity; D, diopter; FSLACS, femtosecond laser assisted cataract surgery; AK, arcuate keratotomy; IOL_t_, toric intraocular lens; WTR, with the rule; ATR, against the rule. The values were expressed as mean±SD or median [Q1, Q3]. F-U, follow-up; BCVA, best corrected visual acuity; LogMAR, logarithm of the minimum angle of resolution; ocular (refractive) astigmatism, OA; Total keratometric astigmatism, CA_tot_; UDVA, uncorrected distant visual acuity.

with-the-rule (67.5 ~ 112.5 = 90 ± 22.5), against-the-rule (0 ~ 22.5 or 157.5 ~ 180), or oblique (intermediate).

§The mean follow-up period was 16.29 ± 11.15 months.

### Visual outcomes

**Figure 1:**
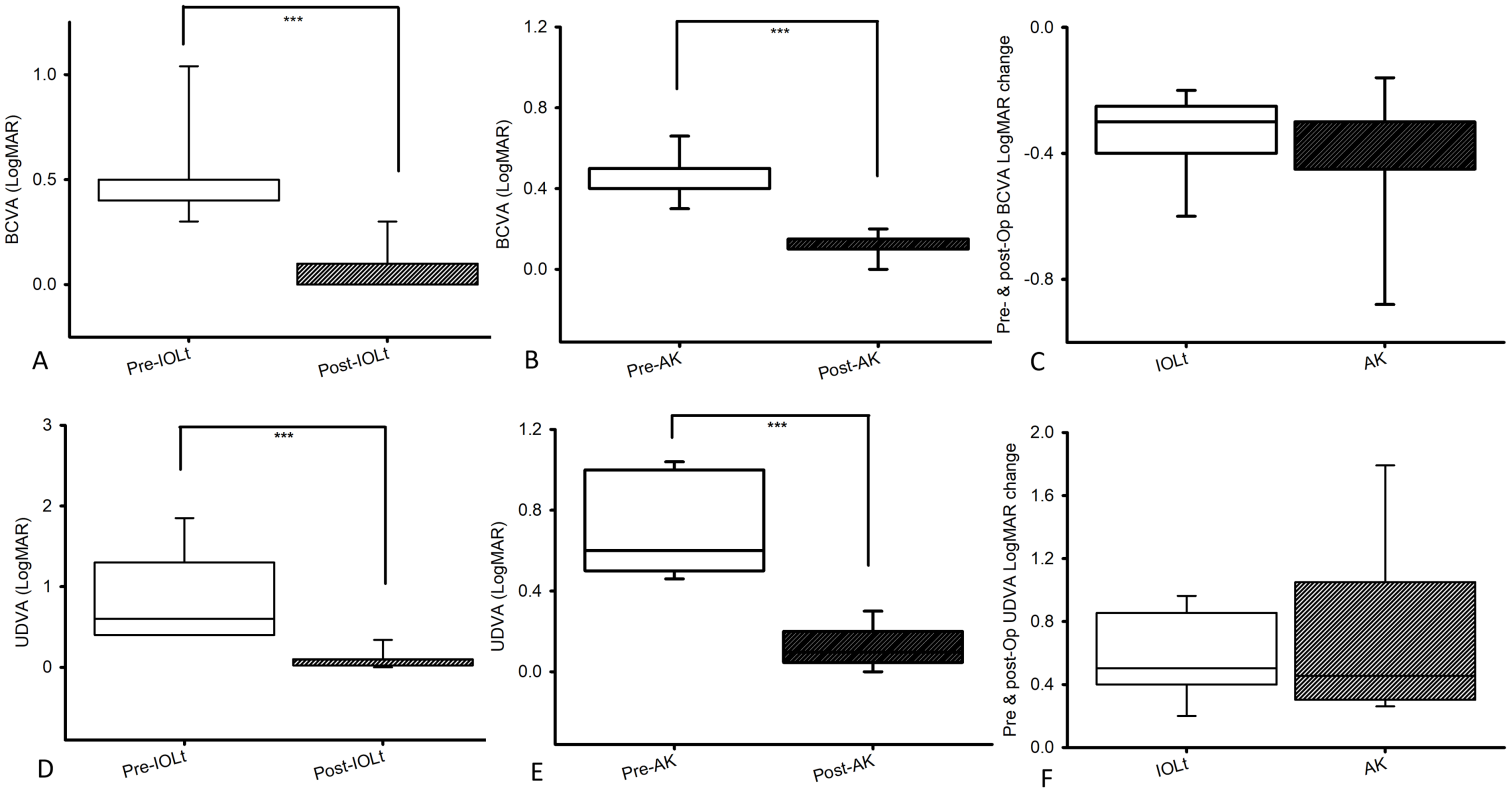
Pre-operative best corrected visual acuities (BCVA; LogMAR) were 0.40 [0.40, 0.50] in IOL_t_ **(A)** and 0.40 [0.40, 0.50] in AK **(B)**, and significantly (*p* < 0.001) improved to 0.10 [0.00, 0.10] in IOL_t_ **(A)** and 0.10 [0.10, 0.15] in AK **(B)** post-operatively. Both pre-operative BCVAs were not significantly (*p* = 0.677) different. The pre-operative and post-operative BCVA alterations were not significantly different between the two groups (**C**; IOL_t_ vs. AK: −0.30 [−0.45, −0.30] vs. − 0.30 [−0.40, −0.25]; *p* = 0.49). On the other hand, pre-operative uncorrected distant visual acuities (UDVA; LogMAR) were 0.60 [0.40, 1.30] in IOL_t_ **(D)** and 0.60 [0.50, 1.00] in AK **(E)**, and significantly (*p* < 0.001) improved to 0.10 [0.02, 0.10] in IOL_t_ **(D)** and 0.1 [0.05, 0.20] in AK **(E)** post-operatively. Both pre-operative UDVAs (LogMAR) were not significantly (*p* = 0.677) different. The pre-operative and post-operative UDVA (LogMAR) changes were not significantly different between the two groups (**F**; IOL_t_ vs. AK: 0.45 [0.30, 1.05] vs. 0.50 [0.40, 0.85]; *p* = 0.85). pre-operative, Pre-Op; AK, arcuate keratotomy; IOL_t_, toric intraocular lens; LogMAR, logarithm of the minimum angle of resolution.

In Figure 1, pre-operative best corrected visual acuities (LogMAR; median [Q1, Q3]) were 0.40 [0.40, 0.50] in IOL_t_ (*n* = 45; A) and 0.40 [0.40, 0.50] in AK (*n* = 15; 1B), and significantly (both *p* < 0.001) increased to 0.10 [0.00, 0.10] in IOL_t_ (*n* = 45; A) and 0.10 [0.10, 0.15] in AK (B) post-operatively. Both pre-operative BCVAs were not significantly (*p* = 0.677) different. The pre-operative and post-operative BCVA alterations were not significantly different between the two groups (C; IOL_t_ vs. AK:-0.30 [−0.45, −0.30] vs. − 0.30 [−0.40, −0.25]; *p* = 0.49). In addition, pre-operative uncorrected distant visual acuities (UDVA; LogMAR) were 0.60 [0.40, 1.30] in IOL_t_ (D) and 0.60 [0.50, 1.00] in AK (E), and significantly (*p* < 0.001) improved to 0.10 [0.02, 0.10] in IOL_t_ (D) and 0.1 [0.05, 0.20] in AK (E) post-operatively. Both pre-operative UDVAs (LogMAR) were not significantly (*p* = 0.74) different. The pre-operative and post-operative UDVA (LogMAR) changes were not significantly different between the two groups (F; IOL_t_ vs. AK: 0.45 [0.30, 1.05] vs. 0.50 [0.40, 0.85]; *p* = 0.85).

### Astigmatism outcomes (OA_tot_, CA_ant_, CA_post_, CA_tot_)

**Figure 2:**
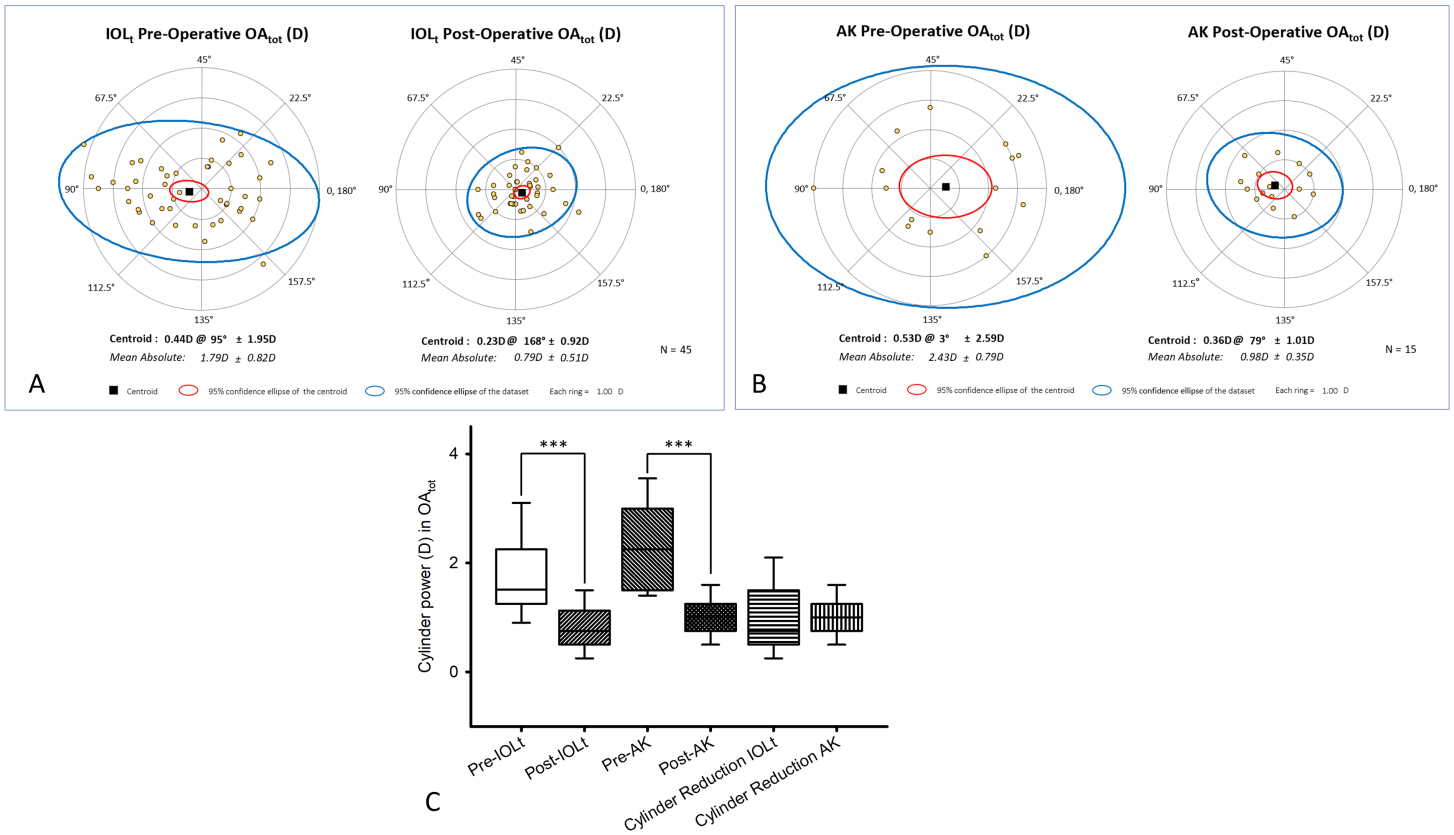
Standard polar plots and cylinder power distribution of total ocular astigmatism (OAtot). Polar plots illustrate the distribution of refractive astigmatism in the **(A)** IOLt group and **(B)** AK group. Each data point represents an individual eye, with the black square indicating the centroid (vector mean) and the red ellipse representing the 95% confidence interval of the centroid. The blue ellipse encompasses 95% of the data points for the entire group. In both cohorts, a statistically significant reduction in OA_tot_ was observed post-operatively (*p* < 0.001). Specifically, the IOLt group (A) centroid shifted from 0.44 D x 95° to 0.23 D x 168°, while the AK group **(B)** centroid shifted from 0.53 D x 3° to 0.36 D x 79°. **(C)** Box-and-whisker plots displayed the distribution of cylinder power (diopter) for both cohorts, showing significant magnitude reduction post-operatively (***p < 0.001), alongside the absolute cylinder reduction achieved in each group. Pre-operative OA_tot_ were 1.51 [1.25, 2.25] D in IOL_t_ and 2.25 [1.50, 3.00] D in AK, and significantly (***p < 0.001) reduced to 0.75 [0.50, 1.13] D in IOL_t_ and 1.00 [0.75, 1.25] D in AK. The pre-operative and post-operative decrease in astigmatism between IOL_t_ (0.75 [0.50, 1.50] D) and AK (1.15 ± 0.211.00 [0.75, 1.25] D) was not significantly (*p* = 0.62) different.

We have demonstrated polar plots that include individual data points, the Centroid (vector mean), and 95% confidence ellipses for both the centroid and the entire dataset. As for OA_tot_, significant reductions were observed in both the IOL_t_ (Figure 2A) and the AK (Figure 2B) groups (*p* < 0.001). In the IOL_t_ group, the mean absolute astigmatism was reduced from 1.79 D ± 0.82 D to 0.79 D ± 0.51 D (centroid, vector mean; centroid shift: 0.44 D x 95 to 0.23 D x 168). In the AK group, the mean absolute astigmatism was reduced from 2.43 D ± 0.79 D to 0.98 D ± 0.35 D (centroid shift: 0.53 D x 3° to 0.36 D x 79°). Box-and-whisker plots (Figure 2C) displayed the distribution of cylinder power for both cohorts, showing significant magnitude reduction post-operatively (***p < 0.001), alongside the absolute cylinder reduction achieved in each group. Specifically, OA_tot_ (Figure 2C; median [Q1, Q3]) fell significantly in both groups, from 1.51 [1.25, 2.25] to 0.75 [0.50, 1.13] D in IOLt and from 2.25 [1.50, 3.00] to 1.00 [0.75, 1.25] D in AK (both *p* < 0.001), which highlights effective astigmatism correction in both groups. The magnitude of reduction (0.75 [0.50, 1.50] vs. 1.00 [0.75, 1.25]) did not differ significantly (*p* = 0.62) in view of correction effectiveness.

**Figure 3:**
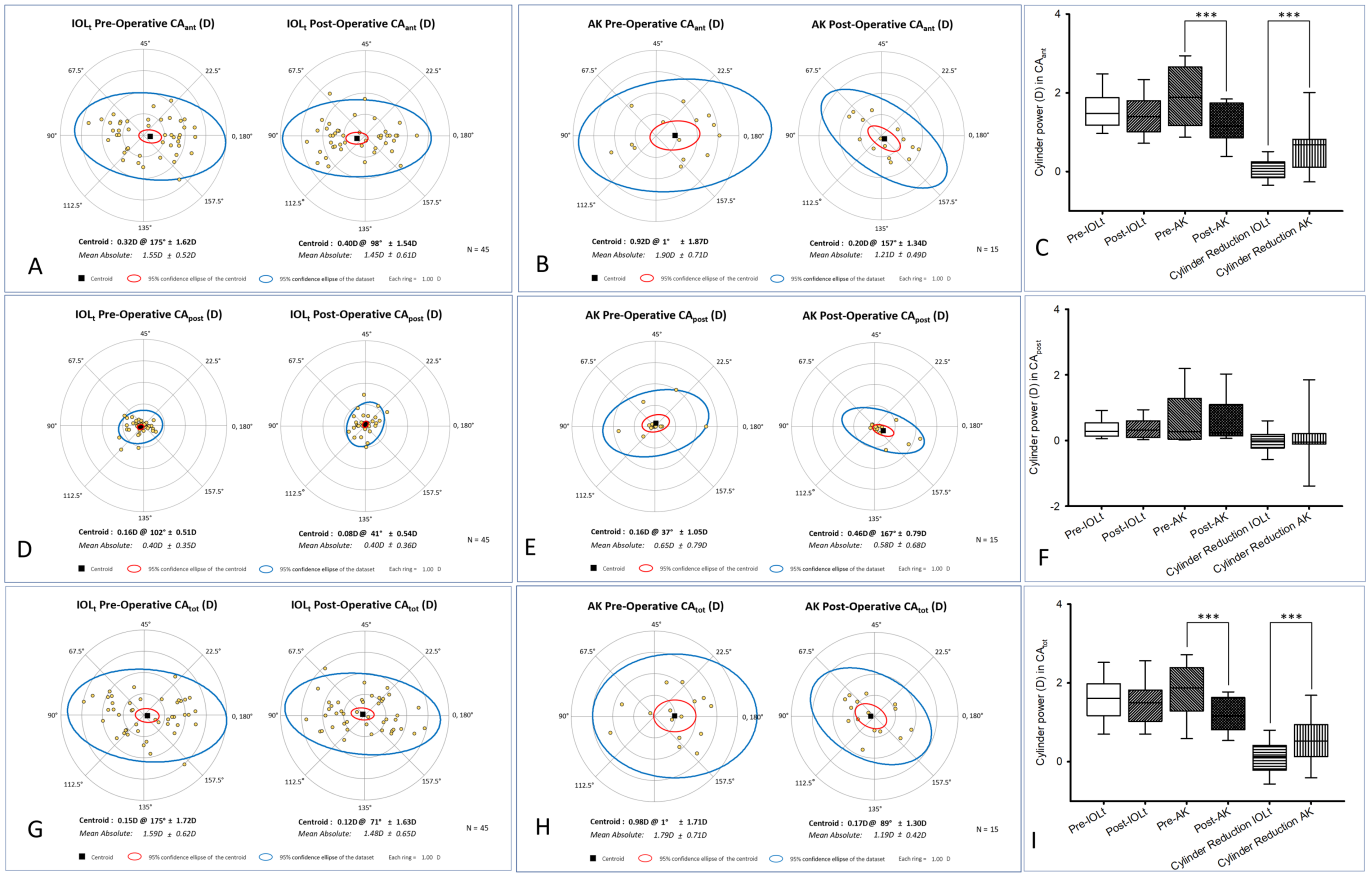
Polar plot and distribution analysis of corneal astigmatism components. This figure compared pre-operative and post-operative corneal astigmatism metrics for the study groups. Anterior corneal astigmatism (CAant) was displayed for the IOLt **(A)** and AK **(B)** groups, with a significant centroid shift noted only in the AK cohort (0.92 D x 1° to 0.20 D x 157°). Posterior corneal astigmatism (CApost) plots for IOLt **(D)** and AK **(E)** demonstrate stability of the posterior corneal surface following both procedures. Total corneal astigmatism (CAtot) for IOLt **(G)** and AK **(H)** groups shows that the AK cohort experienced a significant reduction (*p* < 0.001), with the centroid moving from 0.98 D x 1° to 0.17 D x 89°. Accompanying box-and-whisker plots **(C, F, I)** represent the distribution of cylinder power and the absolute cylinder reduction for CA_ant_, CA_post_, and CA_tot_, highlighting significant changes for CA_ant_ and CA_tot_ primarily within the AK group (***p < 0.001). C. Pre-operative anterior corneal astigmatisms (CA_ant_) were [1.55 (0.53, 0.08) D] in IOL_t_, and not significantly (*p* = 0.43) reduced to 1.45 [0.62, 0.09] D in IOL_t_. In contrast, pre-operative CA_ant_ values [median (Q1, Q3)] were 1.90 [0.74, 0.19] D in AK, and significantly (*p* < 0.001) reduced to 1.22 [0.51, 0.13] D in AK. In terms of CA_ant_, the pre-operative and post-operative reduction in astigmatism between IOL_t_ [0.14 (−0.16, 0.25) D] and AK [0.68 (0.11, 0.82) D] was significantly (*p* = 0.002) different. F. Pre-operative posterior corneal astigmatisms (CA_post_; median [Q1, Q3]) were 0.28 [0.13, 0.54] D in IOL_t_, and not significantly (*p* = 0.80) reduced to 0.32 [0.09, 0.60] in IOL_t_. Likewise, pre-operative CA_post_ values (median [Q1, Q3]) were 0.27 [0.04, 1.28] D in AK, and not significantly (*p* = 0.82) reduced to 0.23 [0.14, 1.10] D in AK. In terms of CA _post_ [median(Q1, Q3)], the pre-operative and post-operative reduction in astigmatism between IOL (*_t_*-0.01 [−0.23, 0.19] D) and AK (0.05 [−0.11, 0.21] D) was not significantly (*p* = 1.00) different. **(I)** Pre-operative total corneal astigmatisms (CA_tot_) were 1.61 [1.17, 1.98] D in IOL_t_, and not significantly (*p* = 0.29) reduced to 1.50 [1.03, 1.82] D in IOL_t_. In contrast, pre-operative CA_tot_ values (mean±SD) were 1.79 ± 0.0.74 D in AK, and significantly (*p* = 0.01) reduced to 1.19 ± 0.43 D in AK. In terms of CA_tot_, the pre-operative and post-operative reduction in astigmatism between IOL_t_ (0.12 ± 0.47 D) and AK (0.59 ± 0.69 D) was significantly (*p* = 0.004) different.

In regard to CA_ant_, a statistically significant change was observed in the AK group (Figure 3B), where the centroid shifted from 0.92 D x 1 to 0.20 D x 157. No significant change (0.32 D x 175 to 0.40 D x 98) was noted in the IOL_t_ group (Figure 3A). Accompanying box-and-whisker plots (Figure 3C) represented the distribution of cylinder power and the absolute cylinder reduction for CA_ant_, highlighting significant changes primarily within the AK group (***p < 0.001). To be specific, for CA_ant_ (median [Q1, Q3]), IOL_t_ (Figure 3C) showed little change (1.55 [0.53, 0.08] to 1.45 [0.62, 0.09] D; *p* = 0.43), whereas AK (Figure 3C) achieved a significant reduction (1.90 [0.74, 0.19] to 1.22 [0.51, 0.13] D; *p* < 0.001). The difference between two groups (Figure 3C) was significant (0.14 [−0.16, 0.25] vs. 0.68 [0.11, 0.82] D; *p* = 0.002). Regarding the correction rate, percentage of eyes achieving ≤0.50 D residual astigmatism was included as follows, i.e., the one of IOLt = 40% (19/45) vs. that of Ak = 20% (3/15). The diffference was not significantly (*p* = 0.13). In term of CA_post_, there was no statistically significant difference in CA_post_ for either the IOL_t_ (Figure 3D; pre-operative 0.1 D x 102 to post-operative 0.08 × 41) or AK (Figure 3E; 0.16 × 37 to 0.46 × 167) groups, indicating stability of the posterior corneal surface. Associated box-and-whisker plots (Figure 3F) demonstrated the values of cylinder power and the absolute cylinder reduction for CA_post_. Specifically, CA_post_ (median [Q1, Q3]) remained unchanged in both groups (IOL_t_: 0.28 [0.13, 0.54] to 0.32 [0.09, 0.60], *p* = 0.80; AK: 0.27 [0.04, 1.28] to 0.23 [0.14, 1.10], *p* = 0.82), and with no significant intergroup difference (−0.01 [−0.23, 0.19] vs. − 0.05 [−0.11, 0.21] D; *p* = 1.00).

As for CA_tot_, the AK group (Figure 3H) showed a significant pre- to post-operative change (*p* < 0.001), with the absolute mean CA_tot_ decreasing from 1.79 D ± 0.71 D to 1.19 D ± 0.42 D. No significance was found in the IOL_t_ group (Figure 3G: 1.59 D ± 0.62 D to 1.48 D ± 0.65 D). Relevant box-and-whisker plots (Figure 3I) revealed the data of cylinder power and the absolute cylinder reduction for CA_tot_, highlighting significant alterations initially within the AK group (***p < 0.001). To be specific, CA_tot_ for IOL_t_ (median [Q1, Q3]) showed no significant change (1.61 [1.17, 1.98] to 1.50 [1.03, 1.82] D; *p* = 0.29), whereas AK (mean±SD) decreased significantly (1.79 ± 0.74 to 1.19 ± 0.43 D; *p* = 0.01). Intergroup comparison (Figure 3I; mean±SD) confirmed a significant difference (0.12 ± 0.47 vs. 0.59 ± 0.69 D; *p* = 0.004).

### Vector astigmatism analysis

**Figure 4:**
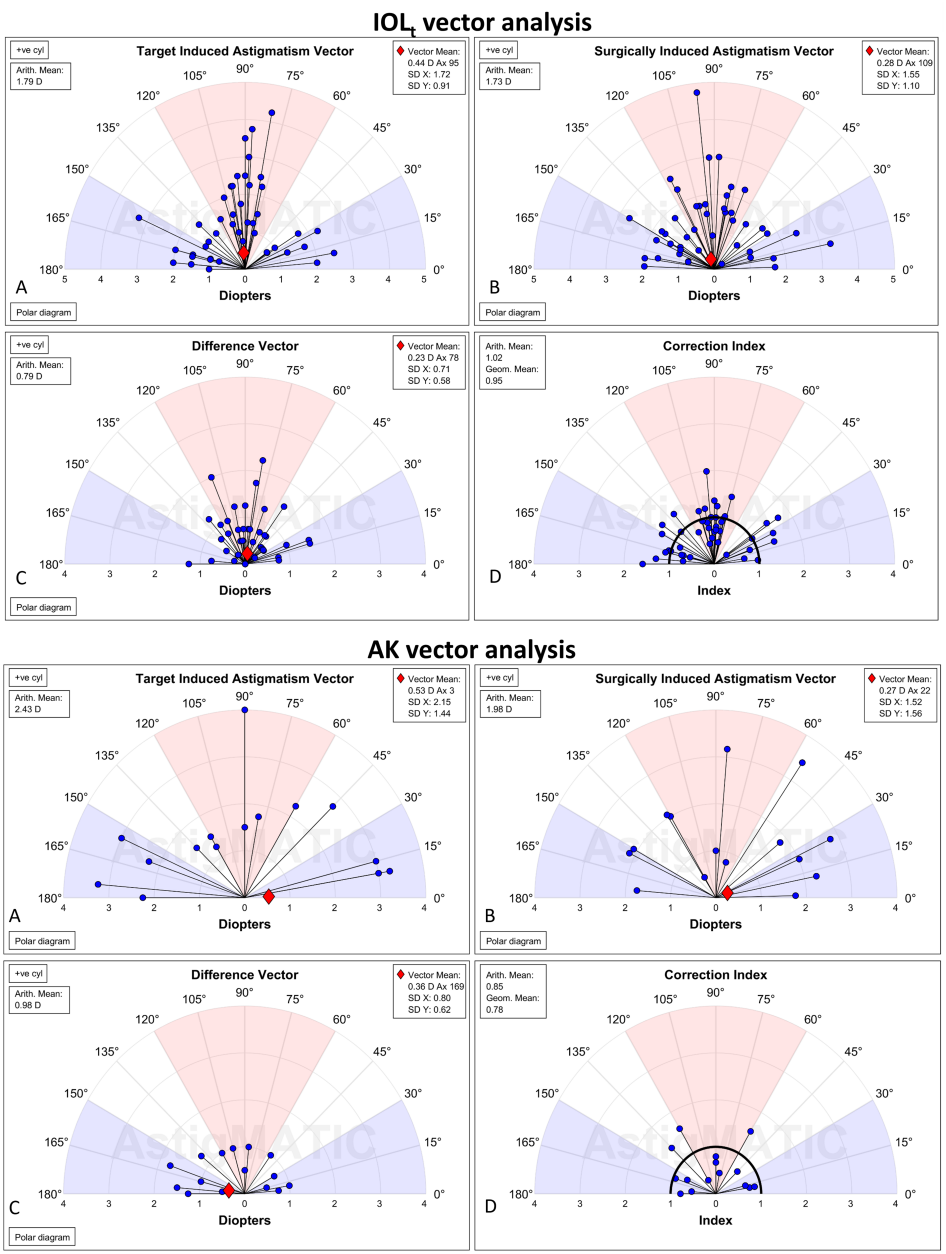
Alpins method vector analysis for the IOLt group. Double angle vector diagrams (DAVD) illustrated the surgical outcomes for the IOLt cohort (*n* = 45). TIA **(A)** showed a mean±SD of 1.79 ± 0.83 D, and SIA **(B)** demonstrated a mean±SD of 1.73 ± 0.80 D. The DV **(C)** was 0.79 ± 0.51 D. The CI **(D)** revealed a mean±SD of 1.02 ± 0.36 indicates a high level of surgical predictability with minimal over or under-correction. **(E–H)** Alpins method vector analysis for the AK group. DAVD stated the operative results for the AK cohort (*n* = 15). TIA (mean±SD; E) was 2.43 ± 0.82 D, while the achieved SIA **(F)** was 1.98 ± 0.81 D. The resulting DV **(G)** was 0.98 ± 0.36 D. The CI (**H**; mean ± SD) of 0.85 ± 0.38 suggests a trend toward slight under-correction in this group compared to the IOLt group.

In view of vector analysis, Figures 4 A–H utilized Double Angle Vector Diagrams to display the distribution of surgical vectors. As shown in Figure 4E: TIA was, respectively, 1.79 ± 0.83 D for the IOL_t_ group and 2.43 ± 0.82 D for the AK group. Moreover, SIA was 1.73 ± 0.80 D for IOLt (Figure 4B) and 1.98 ± 0.81 D for AK (Figure 4F). Furthermore, the DV was limited to 0.79 ± 0.51 D in the IOL_t_ group (Figure 4C) and 0.98 ± 0.36 D in the AK group (Figure 4G). What is more, the IOL_t_ group showed high predictability (CI = 1.02 ± 0.36; Figure 4D), while the AK group showed slight under-correction (CI = 0.85 ± 0.38; Figure 4H).

### Total corneal HOAs

**Figure 5:**
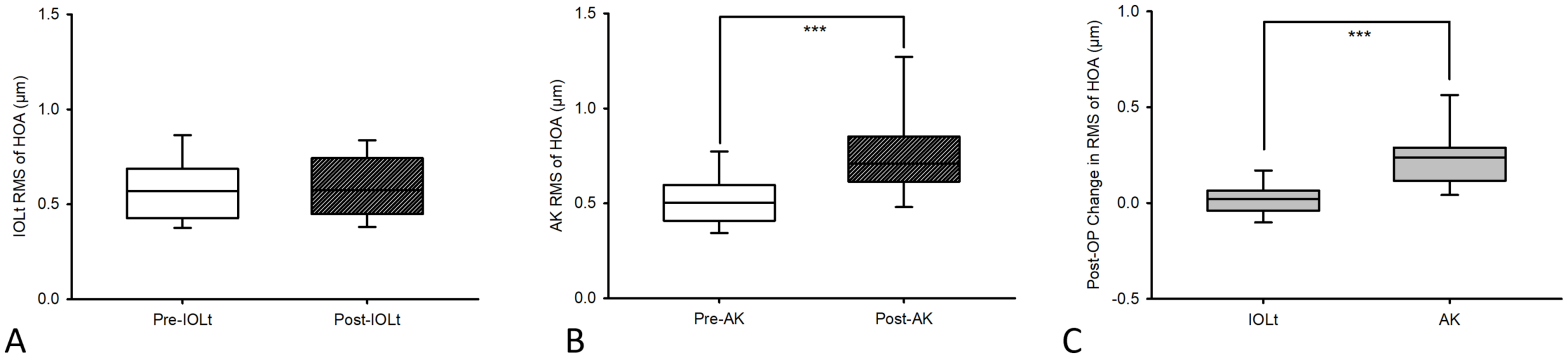
Pre-FSLACS-IOL_t_ [median(Q1, Q3)] total corneal HOAs 0.57 [0.43, 0.69] did not significantly (*p* = 0.71; **(A)** reduce to 0.57 [0.45, 0.74] after FSLACS IOL_t_. However, pre-FSLACS AK total corneal HOAs 0.50 [0.41, 0.60] significantly increased after FSLACS-AK (0.71 [0.61, 0.85]; *p* = 0.003; **(B)**. Furthermore, **(C)**, the pre-operative and post-operative changes in total corneal HOAs (median[Q1, Q3] between IOL_t_ (0.02, [−0.04, 0.06]) and AK (0.24, [0.12, 0.29]) were significantly (*p* < 0.001) different.

HOAs Figure 5; mean [Q1, Q3]) remained stable in IOLt (Figure 5A; pre-IOL_t_ (0.57 [0.43, 0.69] to post IOL_t_ 0.57 [0.45, 0.74]; *p* = 0.71), but increased significantly in AK (Figure 5B; pre-AK (0.50 [0.41, 0.60]) to post-AK (0.71 [0.61, 0.85]); *p* = 0.003). The between-group (IOLt vs. AK) difference in change was significant (Figure 5C; 0.02, [−0.04, 0.06] vs. 0.24, [0.12, 0.29]; *p* < 0.001). This indicates that AK may introduce subtle corneal irregularities, whereas IOLt have demonstrated to maintain stable corneal morphology.

### CDE

**Figure 6:**
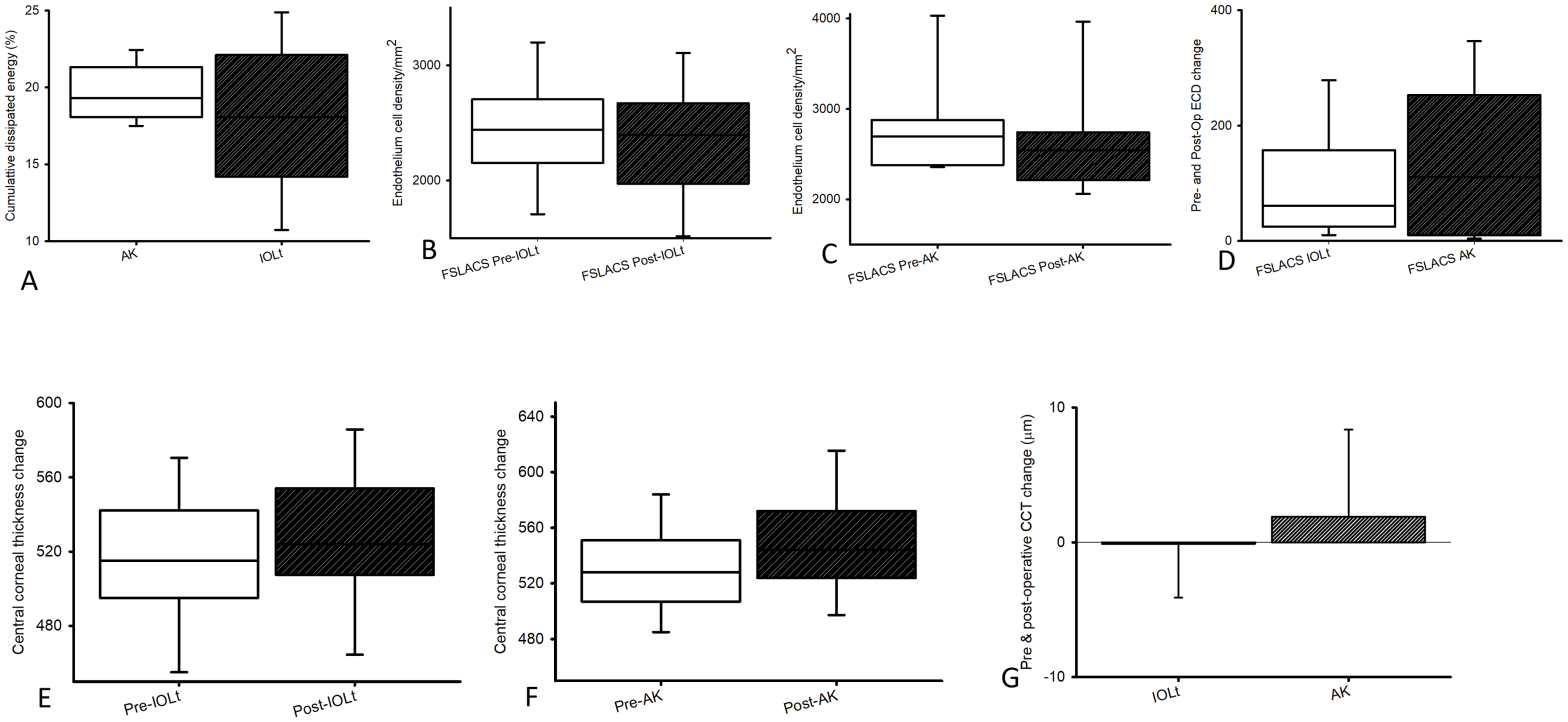
Cumulative dissipated energy (CDE; **A**). The mean CDE values were analyzed for both groups. The difference between Group 1 (FSLACS-IOL_t_; *n* = 45) and Group 2 (FSLACS -AK; *n* = 15) was statistically not significant (*p* = 0.20). ECD **(B–D)**. Prior to the FSLACS-IOL_t_ or FSLACS-AK **(B)**, the ECD was recorded at an average of 2453.10 (550.94, 82.13) (*n* = 45) or 2694.40 [2379.00, 2878.00] cells/mm^2^ (*n* = 15). Following the operation **(C)**, a alteration in ECD was not significantly (*p* = 0.34 or *p* = 0.30) observed, with a post-operative average of 2341.57 [545.68, 81.35] (*n* = 45) or 2540.50 [2211.88, 2740.30] cells/mm^2^ (*n* = 15). In terms of the baseline, there was no significant difference (*p* = 0.55) in the pre-operative ECD between the two groups. Of note **(D)**, there was no significant (*p* = 0.54) difference between the post-operative ECD loss following FSLACS-IOL_t_ (61.30 [24.60, 157.45] cells/mm^2^) and the one after FSLACS AK (110.60 [10.00, 253.20] cells/mm^2^). Central corneal thickness (CCT; **E–G**). **(E)**. In Group 1 (FSLACS IOL_t_: median[Q1, Q3]), mean CCT was 515.00 [495.00, 542.25] μm pre-operatively and 524.00 [507.50, 554.00] μm post-operatively (*p* = 0.06). **(F)**. In Group 2 (FSLACS-AK), CCT was 531.13 ± 32.45 μm pre-operatively and 550.87 ± 39.44 μm post-operatively (*p* = 0.15). (**G**). Change in CCT from baseline [median(Q1, Q3)], Group 1 showed a post-operative change of 10.00 [5.50, 18.50] μm and Group 2 demonstrated a post-surgical alteration of 17.00 [8.00, 31.00] μm; no significant difference was observed between groups (*p* = 0.06).

CDE (Figure 6A; median [Q1, Q3]) was similar between both AK and IOL_t_. Specifically, IOL_t_ cases required 19.30 [18.07, 21.33] and AK cases used 18.07 [14.18, 22.12] units of CDE, which is calculated as a non-significant difference (*p* = 0.20).

### Endothelial cell density

Endothelial health was largely preserved in both groups. ECD (Figures 6B–D; median [Q1, Q3]) fell from 2453.10 [550.94, 82.13] to 2341.57 [545.68, 81.35] cells/mm^2^ in IOL_t_, whereas 2694.40 [2379.00, 2878.00] to 2540.50 [2211.88, 2740.30] cells/mm^2^ in AK (*p* = 0.34 and 0.30). Although, pre-AK ECD values were slightly higher than pre-IOLt ones; yet, both were not significantly different (*p* = 0.08). Moreover, the extent of cell loss (61.30 [24.60, 157.45] vs. 110.60 [10.00, 253.20] cells/mm^2^) did not differ significantly (*p* = 0.54).

Corneal thickness (Figures 6E–G) remained stable in both groups. In IOL_t_ (Figure 6E; median [Q1, Q3]), CCT was 515.00 [495.00, 542.25] μm preoperatively to 524.00 [507.50, 554.00] μm postoperatively (*p* = 0.06). In AK (Figure 6F; mean ± SD), values were 531.13 ± 32.45 to 550.87 ± 39.44 μm (*p* = 0.15). No significant difference (median [Q1, Q3]; IOLt vs. AK = 10.00 [5.50, 18.50] vs. 17.00 [8.00, 31.00] was seen between groups after IOLt or AK (Figure 6G; *p* = 0.06).

## Discussion

This investigation was performed to evaluate whether FSLACS plus AK or IOL_t_ implantation is more suitable for astigmatism correction (6, 7). Astigmatism is known to be associated with decreased BCVA, where an oblique residual astigmatism of 0.5 diopter can potentially lower high-contrast visual acuity by approximately one line on the logMAR scale (5). For correction of very low to moderate astigmatism, AK may be considered as a viable option (6). Furthermore, FSLACS plus AK enables precise and reproducible corneal incisions with predictable outcomes (8). However, the effectiveness of AK may diminish over time due to regression associated with corneal wound healing (9).

In contrast to manual AK, the astigmatic correction achieved by FSLACS plus AK can be adjusted intraoperatively by “titrating” the incision parameters. However, a universally accepted and precise nomogram for FSLACS-AK has yet to be fully established. Regarding IOL_t_ implantation, the anterior corneal astigmatism (CA_ant_), posterior corneal astigmatism (CA_post_), and total corneal astigmatism and CA_tot_) should also be considered when determining nomogram inputs to optimize surgical outcomes (4, 10). In this present investigation, this approach demonstrated stable and sustained outcomes over a follow up period of 14.02 months, via a standardized nomogram (Catalys, Johnson & Johnson) with an 8-mm optical zone and 80% corneal depth.

As mentioned previously, this present study aimed to evaluate the outcomes of FSLACS plus mono-aspheric IOL (ZCB, Johnson & Johnson) together with AK compared to FSLACS plus IOL_t_ (ICB, Johnson & Johnson) in eyes with pre-operative OA_tot_ (up to 3.0 D) and to assess OA_tot_, or CA_tot_/CA_ant_/CA_post_ power before and after FSLACS plus either AK or IOL.

### Similarity or dissimilarity in OA or CA between the present study and previous researches

Our study demonstrated a significant reduction in OA_tot_ after surgery in either FSLACS plus IOL_t_ (FSLACS-IOL_t_) group, or FSLACS plus monoaspheric IOL together with AK (FSLACS-AK) group. Moreover, the FSLACS-AK group showed similar results in OA_tot_ during the correction of low-to-moderate astigmatism comparable to those of the FSLACS-IOLt group. In addition, both groups did not significantly result in significant post-operative ECD reduction and CCT changes. Specifically, the mean OA_tot_ correction was 61% in the FSLACS-IOL_t_ group and 54% in the FSLACS-AK groups, consistent with findings from other comparable studies (11). This supports the effectiveness of both procedures for OA_tot_ correction in cataract surgery. Similar to the present study in FSLACS-AK group (from 2.43 D to 1.13 D: 54% reduction), Yoo et al. reported that refractive astigmatism decreased significantly from 1.71 D to 0.78 D (46% reduction) when penetrating FSLACS-AK was applied to the cornea (diameter = 9.0 mm *vs.* the present 8 mm; depth = 85% *vs.* the present 80%) to correct residual astigmatism after cataract surgery using a 60-kHz IntraLase femtosecond laser. The investigators compared this with IOLt implantation in cataract patients diagnosed astigmatism and, like our study, found no significant difference in residual refractive astigmatism between the two treatment methods, reinforcing the comparable efficacy observed in our investigation (7).

In the present study, FSL FSLACS-AK group was performed using paired arcuate incisions on steep axes, positioned entirely within the corneal stroma, with an arc length of 85 degrees. Not inconsistent with these findings in CA_tot_ (pre-operative 1.79 to postoperative 1.19, i.e., 34% reduction), Day et al. (12) reported a decrease in corneal astigmatism by 39%, from 1.21 D pre-operatively to 0.74 D post-operatively, in a cohort of 196 eyes treated with intrastromal FSLACS-AK.

Using the Catalys FSL system (Catalys; Johnson & Johnson), this present study observed a reduction in OA_tot_ approximately one year after surgery, with a 50% reduction in the FSLACS-IOL_t_ group and a 44% reduction in the FSLACS-AK group as mentioned above. Additionally, mean or median CA_ant_ was reduced by 9% in the IOL_t_ group or by 36% in the AK group. These findings differ slightly from previous reports on CA_ant_ reduction following AK using FSL devices (11). Chan and colleagues, using the VICTUS femtosecond laser system (Bausch & Lomb Inc., Dornach, Germany), reported a 45% reduction in CA_ant_, compared to the 36% reduction observed in the present study, probably due to their slightly different preoperative CA_ant_ average of 1.33 D versus 1.90 D in our cohort (11).

Similar to another study using the LenSx FSL device (Alcon), a 35% CA_ant_ (or CA_tot_) reduction was reported (4). As a comparison, an updated presentation (13) in AK reported a decrease in CA_ant_ values (59%) with a preoperative astigmatism average of 1.62 D using another FSL device (FEMTO LDV Z8 FSL, Ziemer Ophthalmic Systems, Port, Switzerland), the results varied from different machines. In our study, a reduction (non-significant vs. significant) of CA_tot_ values (0.12 in IOL_t_ vs. 0.59 D in AK; 7% vs. 34%) was observed with a preoperative astigmatism average of 1.61 D in IOL_t_ vs. 1.79 D in AK using the current FSL device (Catalys; Johnson & Johnson). Similar post-operative reduction rate was also seen in CA_ant_ (a pre-AK value of 1.79 D and post-AK one of 1.19 D, i.e., 34% reduction) as defined. Catalys FSL system presently significantly reduced/affected OA_tot_ in both groups. In contrast, significant changes in CA_ant_/CA_tot_ values were only observed in FSLACS-AK group 1 year postoperatively, but those in FSLACS-IOL_t_ group were of no significance. These outcomes were comparable to those reported using other femtosecond laser platforms. Variations in reduction rates across studies may be attributed to differences in preoperative astigmatism levels and device-specific parameters.

Our results, similar to a previous study (diameter: inner 12.5 mm/outer 19.8 mm in LenSx, Alcon), may be attributable to the similar but slightly smaller liquid optics interface (LOI) diameter (inner 12 mm/outer 19 mm) attached to the laser system (Catalys; Johnson & Johnson) (14). This feature allows for stabilized vacuum/capture/lock, precise corneal incisions with accurate depth, fewer surrounding corneal tissue/stromal injuries, and fewer corneal edematous changes (13). Moreover, while the observed differences may be attributed to the different FSL devices, variations in nomogram design likely contributed as described. In the present study, the Catalys system (Johnson & Johnson) was employed, and the manufacturer’s FSL protocol was followed, involving AIs placed in the 8 mm OZ with an incision depth set to 80% of corneal thickness. Given inter-study variability, further refinement of nomogram parameters may be warranted. Our results are consistent to other investigations studying the effects of FSLACS-IOL_t_ and FSLACS-AK, demonstrating comparable degrees of astigmatism correction (15) as defined.

### FSLACS-IOL_t_ implantation is effective and safe for preoperative astigmatism in cataract surgery

FSLACS-IOL_t_ is recently accepted to be an effective and safe option for correcting pre-operative astigmatism in cataract surgery. Candidates with corneal pathologies related to morphological abnormalities, such as keratoconus, were excluded from the present study to ensure appropriate patient selection. Furthermore, there were no significant differences between the two groups in terms of CDE, ECD, and CCT as mentioned. As described, astigmatism is known to be associated with decreased BCVA, within expectation, significant reductions in OA_tot_ were associated with significant improvement in BCVAs after FSLACS-AK and FSLACS-IOL_t_. Moreover, TIA or SIA did not significantly differ between two groups. This suggests high surgical accuracy and a well-executed surgical plan.

Evaluation of corneal wavefront revealed a significant increase in RMS HOAs in the FSLACS-AK group compared with the FSLACS-IOL_t_ group. The aberrations observed in the present investigation was consistent with previous reports (15). Consequently, FSLACS-AK may induce visual disturbances such as glare and halos by significantly elevating HOAs relative to FSLACS-IOL_t_. Additionally, the corrective effect of FSLACS-AK on CA_ant_ or CA_tot_ together with HOA was significantly affected than that achieved with IOL_t_ in cases of moderate-to-high astigmatism (≥2.0 D), which aligns with previous studies (15). The notable impact of FSLACS-AK on CA_ant_ and CA_tot_ may relevantly help explain the observed significant increase in HOAs following surgery.

There are two primary types of incisions used in FSLACS-AK. The first is penetrating FSLACS-AK, which involves a full-thickness incision starting from the anterior corneal surface. This technique, employed in the present study, was also used by Noh et al. (2021). One advantage of penetrating FSLAK is that the wound can be fully opened postoperatively if the astigmatic correction proves insufficient. In contrast, intrastromal FSLAK creates an incision confined within the corneal stroma, sparing the Bowman’s layer and epithelium. This approach carries a lower risk of complications such as infection, epithelial ingrowth, or wound gap. In the study of Noh et al. (2021) with penetrating FSLAK, none of these complications were observed during the 3-month observation period; however, longer-term studies are necessary to confirm safety outcomes (15). Currently, there is insufficient evidence to conclude that penetrating FSLACS-AK provides a significantly greater astigmatic correction than intrastromal FSLAK. Variations in incision depth, arc length, and optical zone diameter across studies, along with the limited number of comparative investigations, contribute to this uncertainty (16).

No study to date has directly compared intrastromal FSLACS-AK with IOL_t_. Moreover, the FSLACS-AK group in Yoo et al.’s study consisted of patients with residual astigmatism following cataract surgery, whereas our study included patients without any prior history of cataract surgery. Beyond the investigation by Noh et al. (15), only one retrospective study has compared penetrating FSLACS-AK with IOL_t_ (*n* = 31) (7), reporting no significant difference between the two methods. Of note, as proved by the current research, after FSLACS-AK, the influences on CA_ant_ or CA_tot_, highly probably associated with significantly increased HOAs, was significantly greater than those achieved with IOL_t_ (*n* = 45), which have been consistent with previous study (15). Since significant increase in HOAs might induce unwanted visual disturbance such as halo and glare, the FSLACS-IOL_t_ appears to be effective and safe for pre-operative astigmatism in cataract surgery.

As this was a retrospective study with unequal group sizes, baseline balance was assessed using standardized mean differences (SMDs). Equivalence testing with a predefined margin of ±0.50 D demonstrated that the 95% confidence interval of the intergroup difference extended beyond the equivalence bounds. In addition, the study power (65%–70%) was borderline (<80%), and a moderate baseline imbalance was observed (SMD = 0.66). Therefore, this retrospective study has inherent limitations in supporting statistical “equivalence” between the two groups.

However, as illustrated in Figure 2, the primary purpose of this analysis was to determine whether either post-AK OA_tot_ or post-IOL_t_ OA_tot_ could be significantly reduced, which is clinically meaningful. Futher studies should recruit a larger number of patients in both groups—particularly in the AK group—to achieve higher statistical power (>80%), establish equivalence, and improve baseline balance. Moreover, in the near future investigation, large-scale randomized prospective controlled trials with longer follow-up periods are warranted to provide more definitive evidence.

## Conclusion

IOL_t_ implantation has proven to be an effective, predictable, and safe method for correcting preoperative mild to moderate ocular or corneal astigmatism in cataract surgery, offering short-term refractive and visual outcomes that are comparable, and not inferior, to those achieved with FSLACS-AK. It also provides notable advantages, such as more stable astigmatic correction and significantly fewer surgically induced higher-order aberrations, which may contribute to enhanced visual quality and greater patient satisfaction. While these findings are promising, further research through large-scale, randomized controlled trials with long-term follow-up is needed to fully assess the stability and comparative efficacy of both techniques. Given its minimal postoperative impact on CA_tot_ and CA_ant_, IOL_t_ implantation may be especially suitable for patients with mild to moderate astigmatism, particularly when preserving corneal integrity is a clinical priority. Nonetheless, a better understanding of the availability and appropriate application of FSL and IOL_t_ types/techniques remain essential for optimizing astigmatism management in cataract surgery.

## Data Availability

The raw data supporting the conclusions of this article will be made available by the authors, without undue reservation.
